# Retrospective evaluation of acid–base imbalances, clinicopathologic alterations, and prognostic factors in hospitalized calves with Eimeria-associated diarrhea

**DOI:** 10.3389/fvets.2024.1467583

**Published:** 2025-01-06

**Authors:** Andrea Urgibl-Bauer, Annette Lorch, Dana Badura, Yury Zablotski, Peter D. Constable, Florian M. Trefz

**Affiliations:** ^1^Clinic for Ruminants with Ambulatory and Herd Health Services, Center for Clinical Veterinary Medicine, LMU Munich, Munich, Germany; ^2^Department of Veterinary Clinical Medicine, College of Veterinary Medicine, University of Illinois Urbana-Champaign, Urbana-Champaign, IL, United States

**Keywords:** coccidiosis, hyponatremia, hypochloremia, strong ion difference, dehydration

## Abstract

**Introduction:**

After the neonatal period Eimeriosis is one of the most common causes of large intestinal diarrhea in calves. In contrast to neonatal calves with diarrhea, there are very few reports about the clinicopathological alterations in affected animals, which are mainly based on experimental data. The aim of the present study was therefore to characterize acid–base and related clinicopathologic alterations in calves with Eimeria-associated diarrhea and to identify variables associated with in-hospital mortality.

**Methods:**

Retrospective analysis of clinical and clinicopathologic findings extracted from medical records of 118 calves aged 1 to 5 months admitted to a veterinary teaching hospital.

**Results:**

Severely affected calves were profoundly hyponatremic and hypochloremic, with a strong correlation between plasma sodium and chloride concentrations (Spearman’s *r_s_* = 0.90). Acidemia was found in 57.6% of calves and was associated with hyperphosphatemia, hyper-L-lactatemia, and the presence of unidentified strong ions. Forty-seven calves (39.8%) did not survive to hospital discharge. Classification tree analysis indicated that hospital mortality was associated with plasma ionized calcium concentrations <1.05 mmol/L, initial leukocyte counts >16 × 10^9^ cells/L, and a poor or cachectic body condition. The resulting sensitivity and specificity for predicting non-survival of this model was 59.6 and 90.1%, respectively. In addition to plasma ionized calcium [Odds ratio (OR) = 0.011] and leukocyte concentrations (OR = 1.08), recumbency (OR = 6.1), albumin (OR = 0.90), and decreased strong ion difference (OR = 0.91) were associated with mortality in a second modeling approach (sensitivity 78.7%, specificity 71.8%).

**Conclusion:**

Calves with Eimeria-associated diarrhea can develop profound clinicopathologic derangements. The identified prognostic factors suggest that advanced disease severity, indicated by an inability to stand and reduced body condition, is associated with a lower chance of survival.

## 1 Introduction

During the post-neonatal period, coccidiosis is one of the most common causes of large intestinal diarrhea in calves (1, 2). Bovine coccidiosis is frequently caused by the two species-specific Eimeria-species *Eimeria bovis* and *Eimeria zuernii*, but also by the less pathogenic species *Eimeria alabamensis* that is most commonly associated with outbreaks of the disease in pastured animals (1, 2). Clinical consequences in calves vary depending on the severity of infestation and are typically characterized by diarrhea with feces containing blood and fibrin, tenesmus, weakness and sometimes fever (1–4).

A clear distinction must be made between large intestinal diarrhea caused by *Eimeria* spp. and small intestinal diarrhea in neonatal calves (< 21 days of age), that is usually associated with management factors and single or mixed infections of Rotaviruses, Coronaviruses, *Cryptosporidium* spp. and *Escherichia coli* (5–8). Diarrhea in neonatal calves can result in clinicopathologic derangements including hemoconcentration, azotemia, hypoglycemia, mild to moderate hyponatremia, hyperkalemia, hyper-D- and L-lactatemia, and the development of acidemia due to strong ion (metabolic) acidosis (9–14). The latter is typically characterized by a low strong ion difference due to hyponatremia accompanied by normo- or hyperchloremia and an increase of unmeasured strong ions such as D-lactate (9, 10). D-lactate is a major contributor to increased plasma strong anion concentration that is absorbed from the gastrointestinal tract following bacterial fermentation processes (10, 15). Although disease severity in neonatal calves with diarrhea is theoretically reflected by the degree of metabolic derangements, laboratory data were shown to be of limited value for predicting the outcome of treatment in affected animals (13).

In contrast to neonatal calves with diarrhea, there are only few reports about clinicopathological alterations and their prognostic relevance in older calves with clinical coccidiosis, which are all based on experimentally infected calves (4, 16–22). Available data indicate that moderate to severe hyponatremia is present in severely affected calves but that acid–base balance and hydration status is rarely altered (4, 17, 22–24), which might be related to similar reductions in plasma sodium and chloride concentrations and therefore to minimal decrease of plasma strong ion difference (17, 25).

The aim of the present study was therefore to characterize acid–base and related clinicopathologic changes in critically ill calves with Eimeria-associated diarrhea and to assess whether clinical and laboratory data are associated with outcome.

## 2 Materials and methods

### 2.1 Calves

For the purpose of this retrospective study, the medical records of 210 calves aged between one and 5 months that had a diagnosis of diarrhea and were admitted to the Clinic for Ruminants with Ambulatory and Herd Health Services, LMU Munich, between May 2005 and August 2023 were reviewed. Calves were identified using the clinic’s hospital record database. Calves were selected for inclusion in this study if they were admitted for treatment of diarrhea, had a positive test result for *Eimeria* spp. oocysts on a fecal examination (flotation technique), and if blood gas, electrolyte and clinical biochemical analyses were performed on admission. As in previous studies, diarrhea was defined as a fecal consistency that permitted feces to run through slightly opened fingers (10, 13). General exclusion criteria for calves were a positive test results for BVDV antigen and incomplete datasets regarding blood gas, electrolyte, and biochemical analyses.

### 2.2 Review of medical records

The clinical information retrieved from the medical records included signalment (age, sex, breed), whether the calf was already weaned or not, respiratory and heart rate, rectal temperature in °C, presence or absence of injected scleral blood vessels, hyperemia of mucous membranes, presence of bloody diarrhea, and presence of tenesmus. As in a previous study (13), the following parameters were categorized using 3-point scales on the initial examination at admission: posture (ability to stand, impaired ability to stand, sternal or lateral recumbency), behavior (bright and alert, depressed, apathetic to comatose), suckling reflex (strong, weak, absent), degree of enophthalmos (none, slight to moderate, severe), skin tent duration assessed on the upper eyelid (normal, slightly to moderately increased, markedly increased), and body condition (good to moderate, poor, cachectic). Also as in previous studies (13, 26), a diagnosis of systemic inflammatory response syndrome on hospital admission was made if two of the following criteria were fulfilled: presence of an abnormal leukocyte count (< 5 or > 12 × 10^9^ cells/L), abnormal rectal temperature (< 38.5°C or > 39.5°C), tachycardia (> 120 beats per minute), and tachypnea (> 36 breaths per minute). Information regarding therapeutic measures, presence of concurrent health problems during the first 48 h of hospitalization, outcome of therapy, post-mortem findings, and the stated reasons for euthanasia was also extracted.

### 2.3 Laboratory analyses

Blood samples were collected from the jugular vein on admission to the hospital before any treatment was administered. Lithium-heparinized blood samples were anaerobically collected using a 2-mL polypropylene syringe and blood pH, pCO_2_, and concentrations of plasma sodium (*c*Na^+^), chloride (*c*Cl^−^), potassium (*c*K^+^), and ionized calcium (*c*Ca^2+^) were determined using blood pH, gas, and electrolyte analyzers (Rapidlab 865 [2005–2012], Rapidpoint 405 [2012–2020], and Rapidpoint 500 [2020–2023], Siemens Healthcare Diagnostics Inc., Tarrytown). Blood pH and pCO_2_ were corrected for rectal temperature using the same equation for all three analyzers.

Automatic analyzers were used for additional serum and plasma biochemical analyses (Hitachi 911 [from 2005–2012] and Cobas c311 [from 2012–2023] analyzers, Roche Diagnostics, Mannheim, Germany). Serum samples (plain tubes) were assayed for concentrations (*c*) of urea (urease), creatinine (picric acid), total protein (biuret), albumin (bromcresol green), and inorganic phosphorus (molybdenum). Blood samples containing lithium heparin and potassium fluoride as a glycostatic agent were analyzed for plasma concentrations of L-lactate (lactate oxidase), glucose (hexokinase) and in some single calves for D-lactate using a D-lactate dehydrogenase assay (27). In 16 calves, serum samples, that had been stored at −25°C up to 3 years, were additionally analyzed for D-lactate (using the same D-lactate dehydrogenase assay) and ß-hydroxybutyrate (hydroxybutyrate dehydrogenase) concentrations. Fecal samples were analyzed for Eimeria oocysts using the flotation technique. Between 2005 and 2012, EDTA blood samples were routinely tested for BVDV-antigen in all hospitalized calves (PCR technique), which was stopped thereafter due to major advances in the national BVDV control program.

### 2.4 Calculations and definitions

Application of the Henderson-Hasselbalch acid–base model included assessment of actual bicarbonate concentration (*c*HCO_3_^−^) and base excess of blood (abbreviated as BE(B), also called *in vitro* base excess) which were automatically calculated by the blood gas analyzers based on previously reported algorithms (10, 13). Anion gap (AG) was calculated in order to quantify the unmeasured anion concentration in plasma based on the following equation (28):


(1) $$AG=\left(cNa^{+}+cK^{+}\right)-\left(cCl^{-}+cHC{O_{3}}^{-}\right)$$

The simplified quantitative physiochemical strong ion approach (29) was additionally applied as it provides a more mechanistic approach to acid–base balance and consequently a more comprehensive assessment of the acid–base status of calves than it is the case for the Henderson-Hasselbalch acid–base model (30, 31). This included calculation of the measured strong ion difference obtained from five strong ions (SID_5_, mEq/L) using the measured value for [Ca^2+^] determined by ion-selective potentiometry and assigning a charge of −1 to L-lactate assuming 100% dissociation such that (31):


(2) $$SID_{5}=cNa^{+}+cK^{+}+cCa^{2+}-cCl^{-}-\mathrm{cL}-\mathrm{lactat}e^{-}$$

To be able to assess the relative importance of sodium and chloride imbalances on acid–base status, the strong ion difference calculated from the plasma concentrations of sodium, potassium and chloride (SID_3_; mEq/L) was additionally obtained as follows (9):


(3) $$SID_{3}=cNa^{+}+cK^{+}-\;cCl^{-}$$

An estimate of the concentration of non-volatile weak acids (A_tot_; consisting of albumin, globulins, and dihydrogen phosphate) in mmol/L was estimated from serum concentrations of total protein as previously described (9):


(4) $$A_{\mathrm{tot}}=0.343\times \mathrm{ctotal}\;\mathrm{protein}$$

The anion charge of non-volatile weak acids (A^−^) was calculated using the experimentally determined value for the negative logarithm of dissociation constant of plasma nonvolatile weak acids (pK_a_ = 7.08) and the following equation (9):


(5) $$A^{-}=\left[\mathrm{Atot}/\left(1+10^{\left(7.08-pH\right)}\right)\right]$$

The unmeasured strong ion concentration was obtained by calculating the strong ion gap (SIG) in mEq/L, defined as the difference between the plasma concentration of unmeasured strong cations and unmeasured strong anions. In contrast to the traditional anion gap (Equation 1), this approach excludes the effect of the anion charge of non-volatile weak acids, whereby (9):


(6) $$SIG=A^{-}–AG$$

Calculated values for SIG were also corrected for the measured concentrations of L-lactate and Ca^2+^ to obtain the concentration of still unidentified strong ions (USI) in mEq/L for the calculation of the effective strong ion difference (SID_eff_) such that (31):


(7) $$USI=SIG+\mathrm{cL}-\mathrm{lactat}e^{-}–cCa^{2+}$$

(8) $$SID_{\mathrm{eff}}=SID_{5}+USI=SID_{3}+SIG$$

Measured concentrations of plasma ionized calcium were corrected for changes in pH from 7.40 by use of the following formula (32):


(9) $$cC{a^{2+}}_{7.40}=cCa^{2+}\times 10^{-0.23\times \left(7.40-pH\right)}$$

Furthermore, a potassium to sodium ratio was calculated by dividing *c*K^+^ with *c*Na^+^ and multiplying values with 100 (33).

In order to detect disproportional changes of plasma chloride concentrations with regards to changes of plasma sodium concentrations, a corrected plasma chloride concentration (*c*Cl_c_^−^) was calculated (34, 35) using the following formula, where normal *c*Na^+^ is the midpoint (142 mmol/L) of the reference interval for plasma sodium concentration:


(10) $$cCl_{c}^{-}=\left(\mathrm{normal}\;cNa^{+}/\mathrm{measured}\;cNa^{+}\right)\times \mathrm{measured}\;cCl^{-}$$

### 2.5 Outcome of treatment

Positive outcome of treatment (PO; survival) was defined as discharge from the hospital, whereas a negative outcome (NO; non-survival) was defined as death or euthanasia during hospitalization.

### 2.6 Statistical analyses

Statistical analyses were performed using SPSS for windows (version 26.0, IBM Corp., Armonk, New York, United States), GraphPad Prism (version 10.1.1, GraphPad software) and the software package R (version 4.3.2., R Core Team). Normality of data was assessed based on results of a Shapiro–Wilk test and visual examinations of QQ plots. As most of the data were not normally distributed, non-parametric tests were used and data are presented as medians and interquartile ranges (Q_1_/Q_3_).

Spearman′s correlation coefficients (*r_s_*) were calculated in order to assess potential associations between parameters. Stepwise forward variable selection for linear regression models were additionally constructed in order to predict venous blood pH and actual bicarbonate concentration by variables of clinical pathology and independent variables of the strong ion difference acid–base model (SID, A_tot_, pCO_2_). For the latter, regression models were calculated twice based on SID_3_ and SIG as well as SID_5_ and USI as representatives of the effective strong ion difference. The relative importance of the included variables was assessed by the order of entry into the model as well as by the change in the model *R*^2^ value (Δ*R*^2^). In case of close associations between independent variables (*r_s_* > 0.70 or < −0.70), only one variable was entered into the model in order to minimize potential effects of collinearity (10). Standardized residual plots of multivariable models were examined to confirm an approximately normal distribution of residuals. Multicollinearity of the final predictors was assessed by calculation of the variance inflation factor; multicollinearity is considered a concern if the variance inflation factor is higher than 10 (36).

Mann–Whitney U-tests were used for pair-wise comparison of continuous variables. Differences between different categories of posture/ability to stand were assessed using a Kruskal-Wallis test. Univariable associations between clinical and laboratory variables and the outcome of therapy was assessed using binary logistic regression analysis, which included calculation of odds ratios (OR) with 95% confidence intervals (95% CI). For multivariable modelling of prognostic factors, predictors with a *p*-value ≤0.2 were further processed via a novel *“best subset”—automated model selection and multimodel inference* approach. This approach, implemented in the “glmulti” statistical package (37), finds the best set of models amongst all possible models. Specifically, best subset approach evaluated 42,152 models resulting from various combinations of five predictors and an additional 1,450 models generated from all possible pairwise interactions between 20 predictors. The “best subset” approach ranked these models based on their Akaike Information Criterion (AIC) values, identifying the optimal model. Subsequently, we compared this model’s AIC with that of the “final” model obtained through a backwards-selection approach, aiming to contrast the two variable selection methodologies, and since the final model of the best subset has a significantly better AIC (AIC difference > 2) as compared to the final model resulting from the backwards selection, the best subset approach was opted for. The fit of the final multivariable regression model was evaluated by means of the Hosmer-Lemeshow goodness-of-fit test. The final multivariable models was also assessed for potential confounding effects (38) by the presence of concurrent health problems.

Data were additionally analyzed using classification tree analysis to identify clinically relevant cut-points as potential mortality predictors. The predictive ability of the models was assessed by calculating the area under the receiver-operating characteristic (ROC) curve and the sensitivity, specificity, positive predictive value (PPV), and negative predictive value (NPV) at the optimal cut-point of predicted probabilities identified on the basis of the Youden index. *p*-values <0.05 were declared as statistically significant, while *p*-values between 0.05 and 0.1 were treated as tendentially significant for all analyses.

## 3 Results

### 3.1 General conditions

A total of 136 calves met the inclusion criteria for the study out of 210 calves aged between one and 5 months that had a record of diarrhea in the clinic’s hospital record database during the elected study period. Of those 136 calves, four calves were excluded due to a positive BVDV test result, 13 calves were excluded due to an incomplete dataset regarding laboratory variables, and one calf that was presented in an agonal state was excluded due to documentation issues. Therefore, a total of 118 calves was used for the analysis reported here. The study population consisted of German Fleckvieh (*n* = 112; 94.9%) and Braunvieh calves (*n* = 6; 5.1%). The median age (Q_1_/Q_3_) was 8.4 (6.6/11.0) weeks, and 23 calves (19.5%) were already weaned on admission to the hospital. The proportion of female and male calves was 65.3% (*n* = 77) and 34.7% (*n* = 41), respectively.

### 3.2 Clinical presentation

Most calves were presented in a critical condition. The majority of calves had bloody diarrhea (*n* = 95; 80.5%) and exhibited tenesmus (*n* = 66; 55.9%). A rectal prolapse was documented in 6 calves on admission. Clinical dehydration (increased duration of skin tenting or presence of enophthalmos) was documented in 97 calves (82.2%). Alterations of behavior and posture/ability to stand were documented in 72 calves (61%) and 50 calves (42.4%), respectively. At least one concurrent health problem was documented during the first 48 h of hospitalization in 31 calves (26.3%), which included pneumonia (*n* = 10), lice infestation (*n* = 6), clinical signs of acute abdomen (*n* = 4), left-sided displacement of the abomasum (*n* = 3), navel infections (*n* = 3), septic arthritis (*n* = 2), and miscellaneous problems (*n* = 4).

### 3.3 Findings of acid–base, electrolyte and biochemical analyses

**Figure 1:**
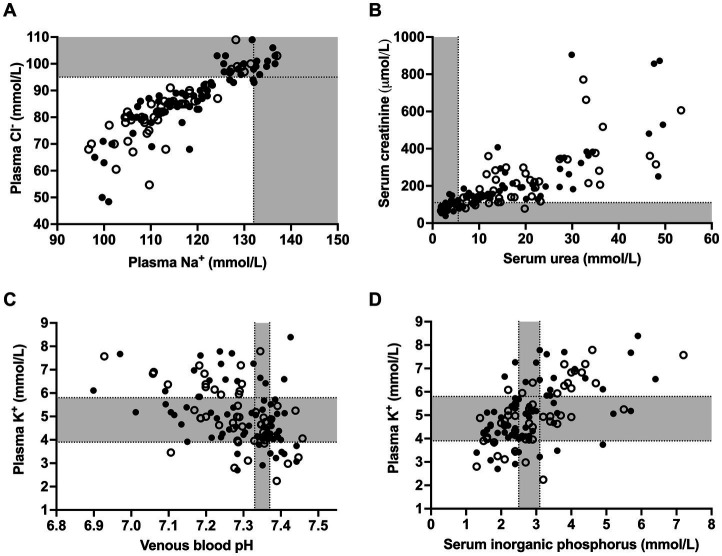
Scatterplots illustrating individual values for plasma sodium and chloride concentrations **(A)**, serum urea and creatinine concentrations **(B)** as well as the association of venous blood pH **(C)** and serum inorganic phosphorus **(D)** with plasma potassium concentrations in 118 calves (1–5 months of age) with Eimeria-associated diarrhea. Closed circles indicate calves with a positive outcome whereas open circles indicate calves that died or were euthanized during hospitalization. Gray-shaded areas represent the reference range of respective variables.

Median and interquartile ranges for selected acid–base and clinicopathologic variables and respective reference ranges are presented in Table 1. The most common clinicopathologic abnormalities included hyponatremia (*n* = 107; 90.7%), hypochloremia (*n* = 90; 76.3%), azotemia (*n* = 82; 69.5%), hyper-L-lactatemia (*n* = 73; 61.9%), and hyperglycemia (*n* = 84; 71.2%). Out of the 107 hyponatremic calves, 61 calves had plasma sodium concentration between 105 and 120 mmol/L, and 14 calves <105 mmol/L. Hyponatremia was accompanied by hypochloremia in 89 calves. As shown in Figure 1A, plasma sodium and chloride concentrations were strongly correlated (*r_s_* = 0.90; *p* < 0.001). Sodium corrected plasma chloride concentrations (calculated using Equation 10) were below and above the reference range in 11 and 12 calves, respectively. Individual values for serum urea and creatinine concentrations are shown in Figure 1B.

**Table 1:** Median and interquartile ranges (Q_1_/Q_3_) of selected laboratory variables in 118 hospitalized calves (1–5 months of age) with Eimeria-associated diarrhea.

Variable	$\tilde{x\;}$	Q_1_/Q_3_	Reference range (9, 27, 41, 53, 101–103)
Henderson-Hasselbalch acid–base model			
pH	7.30	7.21/7.36	[7.33 to 7.37]
pCO_2_ (mm Hg)	44.2	40.4/48.5	[43.5 to 54]
pO_2_ (mm Hg)	37.5	31.4/42.4	[36 to 46.5]
HCO_3_^−^ (mmol/L)	20.9	15.7/26.0	[23 to 29]
BE(B) (mmol/L)	−5.0	−11.5/0.9	[−3.5 to 3.5]
AG (mEq/L)	14.4	10.1/20.1	[8.9 to 15]
SID acid–base model			
A_tot_ (mmol/L)	19.2	16.4/21.5	[15.9 to 21.2]
A^−^ (mEq/L)	11.7	10.3/12.7	n.a.
SID_3_ (mEq/L)	36.0	33.5/38.5	[38.3 to 47.7]
SID_5_ (mEq/L)	35.9	32.9/39.0	[39.9 to 49.7]
SID_eff_ (mEq/L)	32.5	27.2/36.7	[37.3 to 51.5]
USI (mEq/L)	−2.1	6.4/1.2	[−2 to 0]
SIG (mEq/L)	−2.9	−7.9/1.3	[−3 to 3]
Electrolytes			
Na^+^ (mmol/L)	115.6	108.7/125.8	[132 to 152]
K^+^ (mmol/L)	4.9	4.1/6.1	[3.9 to 5.8]
Cl^−^ (mmol/L)	85.5	80/94	[95 to 110]
Cl_c_^−^ (mmol/L)	105	102/108	[95 to 110]
Ca^2+^ (mmol/L)	1.12	1.07/1.19	[1.17 to 1.37]
Ca^2+^_7.40_ (mmol/L)	1.05	0.99/1.14	n.a.
Biochemical analysis			
D-lactate (mmol/L)^1^	0.3	0.19/0.55	[≤ 4.0]
L-lactate (mmol/L)	2.8	1.6/4.3	[≤ 2.2]
Glucose (mmol/L)	6.9	5.4/8.7	[2.3 to 5.8]
ß-Hydroxybutyrate (mmol/L)^1^	0.12	0.09/0.20	[≤ 1.0]
Total protein (g/L)	55.9	47.8/62.6	[59 to 70]
Albumin (g/L)	30.2	27.3/34.6	[30 to 40]
Globulin (g/L)	25.5	21.1/28.9	[30 to 40]
Phosphorus (mmol/L)	2.8	2.2/3.5	[2.5 to 3.1]
Urea (mmol/L)	13.8	6.4/23.5	[≤ 5.5]
Creatinine (μmol/L)	156	109/268	[≤ 110]
Hematologic analysis			
PCV (%)	37.7	32.8/45.3	[30 to 40]
Hemoglobin (g/dL)	11.8	10.3/ 13.9	[10 to 14]
Leukocytes (G/L)	10.9	8.2/15.4	[8 to 12]
Thrombocytes (G/L)	789	629/1167	[200 to 800]

1 Information on D-lactate and ß-hydroxybutyrate concentration were available for 27 and 14 calves, respectively.

$\tilde{x\;}$ (Q_1_/Q_3_), Median and respective interquartile range; n.a., not available; pCO_2_, partial pressure of carbon dioxide; pO_2_, partial pressure of oxygen; BE(B), base excess of blood (in vitro base excess); AG, anion gap; A_tot_, concentration of non-volatile weak acids; A^−^, anion charge of non-volatile weak acids; SID_3_, strong ion difference calculated from three strong ions; SID_5_, strong ion difference calculated from five strong ions; SID_eff_, effective strong ion difference; USI, unidentified strong ions; SIG, strong ion gap; Cl_c_^−^, chloride concentration corrected for change of sodium concentration; PCV, packed cell volume. Variables of the SID acid-base model were calculated by use of Equations 2–8.

Higher than normal serum inorganic phosphorus concentrations (>3.1 mmol/L) were present in 40 calves (33.9%). Hyperkalemia (plasma potassium >5.8 mmol/L) was present in 34 calves (28.8%), whereas hypokalemia (plasma potassium <3.9 mmol/L) was present in 17 calves (14.4%). Plasma potassium concentrations were most closely correlated to serum creatinine (*r_s_* = 0.57; *p* < 0.001) and inorganic phosphorus (*r_s_* = 0.56; *p* < 0.001) concentrations, respectively. The coefficient of correlation between plasma potassium and venous blood pH was (*r_s_* = −0.44; *p* < 0.001). Scatterplots illustrating the association between plasma potassium concentrations, venous blood pH and serum inorganic phosphorus concentrations are shown in Figures 1C,D. Plasma potassium and sodium concentrations, as well as the potassium to sodium ratio was associated with alterations of posture/ability to stand (*p* < 0.001). Recumbent calves had significantly (*p* < 0.001) lower median *c*Na^+^ (108.7 mmol/L), higher *c*K^+^ (6.6 mmol/L), and a higher K^+^/Na^+^-ratio (5.9) than calves with unaltered posture/ability to stand (*c*Na^+^: 120.8 mmol/L; *c*K^+^: 4.6 mmol/L; K^+^/Na^+^-ratio: 3.8).

Acidemia (venous blood pH < 7.33) was present in 68 calves (57.6%), whereas alkalemia (venous blood pH > 7.37) was present in 27 calves (22.9%). Use of the Henderson-Hasselbalch approach for assessment of acid–base balance (by evaluating pCO_2_, *c*HCO_3,_ and AG) indicated the presence of an acid–base derangement in 96 calves (81.4%), of which 68 calves were suffering from a mixed acid–base disorder (i.e., presence of more than one abnormality). Metabolic acidosis (*c*HCO_3_^−^ < 23 mmol/L) was present in 71 calves (60.2%), whereas metabolic alkalosis (*c*HCO_3_^−^ > 29 mmol/L) was present in 14 calves (11.9%). A venous pCO_2_ acidosis (pCO_2_ > 54 mm Hg) was present in 10 calves (8.5%), whereas a venous pCO_2_ alkalosis (pCO_2_ < 43.5 mm Hg) was present in 54 calves (45.8%). Increased values for AG (> 15 mEq/L) were detected in 54 calves (45.8%). Of the 71 calves with metabolic acidosis, 49 calves had increased anion gap (69%), 48 calves had decreased venous pCO_2_ (67.6%) and one calf had increased venous pCO_2_ (1.4%).

Use of the simplified strong ion approach to evaluating acid–base balance (by evaluating pCO_2_, A_tot_, SID_3_, and SIG) indicated the presence of an acid–base derangement in 115 calves (97.5%), of which 89 calves had a mixed acid–base disorder. A strong ion (SID_3_) acidosis (SID_3_ < 38.3 mEq/L) was present in 86 calves (72.9%), whereas a strong ion (SID_3_) alkalosis (SID_3_ > 47.7 mEq/L) was present in 7 calves (5.9%). A buffer ion acidosis (A_tot_ > 21.2 mmol/L) was present in 34 calves (28.8%), whereas a buffer ion alkalosis (A_tot_ < 15.9 mmol/L) was detected in 23 calves (19.5%). A total of 59 calves (50.0%) had increased strong ion gap (< −3 mEq/L). Increased concentrations of unmeasured strong ions (USI ≤ 2 mmol/L) were present in 60 calves (50.8%). Among the 59 calves with increased SIG, 54 (91.5%) had also increased USI concentrations.

### 3.4 Association between clinicopathologic findings and acid–base variables

**Figure 2:**
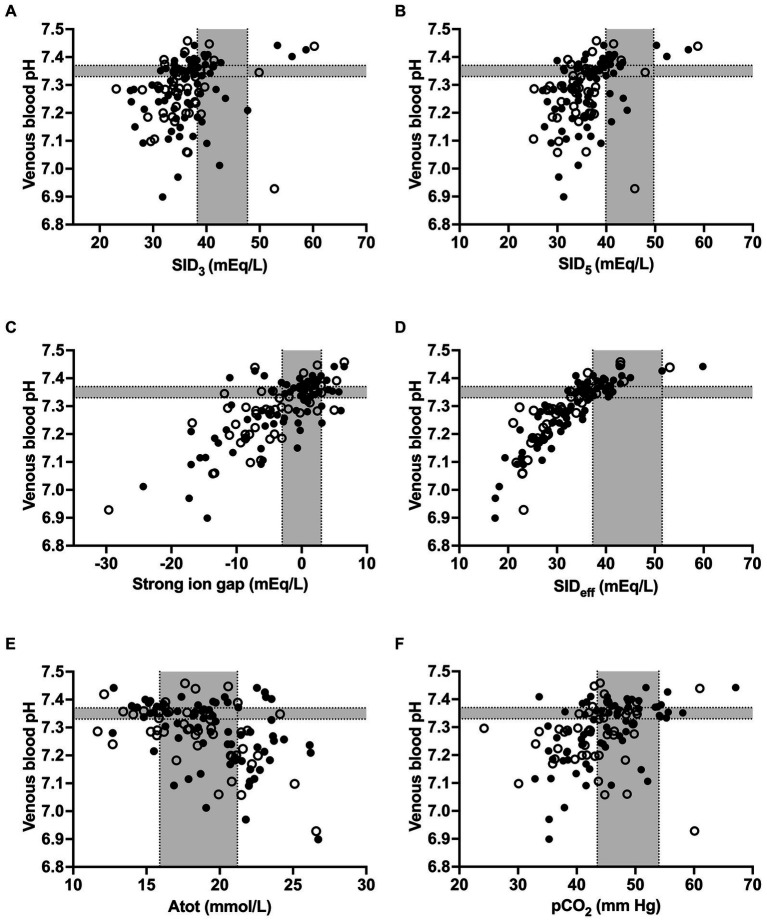
Scatterplots illustrating the association between venous blood pH and strong ion difference calculated from plasma Na^+^, K^+^, and Cl^−^ concentrations **(A)**, strong ion difference calculated from plasma Na^+^, K^+^, Ca^2+^, Cl^−^, and L-lactate^−^ concentrations **(B)**, strong ion gap **(C)**, effective strong ion difference **(D)**, concentration of non-volatile weak acids **(E)**, and partial pressure of carbon dioxide **(F)** in calves of the study population. Closed circles indicate calves with a positive outcome, whereas open circles indicate calves that died or were euthanized during hospitalization. Gray-shaded areas represent the reference range of respective variables.

**Figure 3:**
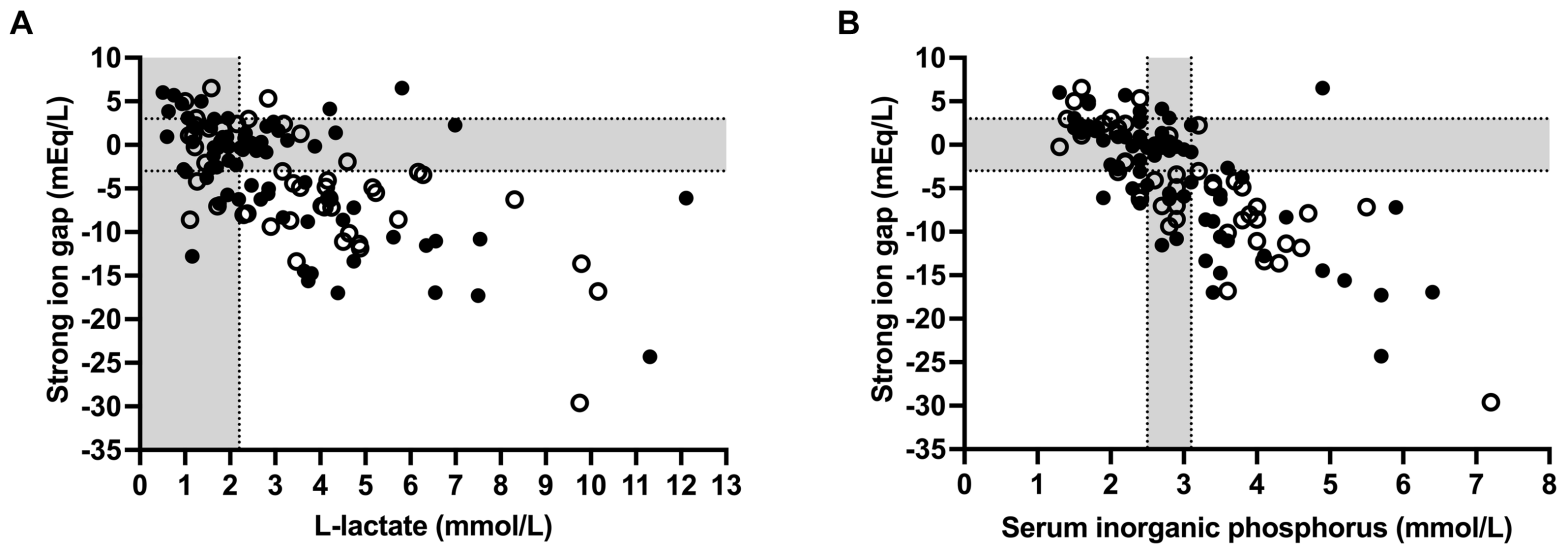
Scatterplots illustrating the association of plasma L-lactate **(A)** and serum inorganic phosphorus concentrations **(B)** with strong ion gap in 118 calves (1–5 months of age) with Eimeria-associated diarrhea. Closed circles indicate calves with a positive outcome, whereas open circles indicate calves that died or were euthanized during hospitalization. Gray-shaded areas represent the reference range of respective variables.

Spearman’s coefficients of correlation between clinicopathologic and acid–base variables are reported in Supplementary Table S1. Scatterplots illustrating the association between venous blood pH and SID_3_, SID_5_, SIG, SID_eff_, A_tot_, and pCO2 as independent variables of the simplified strong ion acid–base model are shown in Figure 2. The coefficients of correlation between venous blood pH and SID_3_, SID_5_, SIG, SID_eff_, A_tot_, and pCO2 were 0.33, 0.48, 0.67, 0.89, −0.43, and 0.45, respectively. Results of a stepwise forward linear regression analysis which aimed to predict venous blood pH and actual bicarbonate concentration by means of independent variables of the strong ion difference approach are reported in Table 2. Partial pressure of carbon dioxide was the most important predictor for actual bicarbonate concentration, whereas venous blood pH was best predicted by SIG and USI, respectively. As shown in Figure 3, SIG was associated with plasma L-lactate (*r_s_* = −0.59) and serum inorganic phosphorus concentrations (*r_s_* = −0.73).

**Table 2:** Results of a stepwise linear regression analysis for predicting venous blood pH and actual bicarbonate concentration (dependent variables) by means of independent variables of the strong ion difference acid–base model based on measured strong ion difference (SID_3_ and SID_5_), unmeasured strong ion difference (SIG and USI), the concentration of non-volatile weak acids (A_tot_), and partial pressure of carbon dioxide (pCO_2_) in 118 calves (1–5 months of age) with Eimeria-associated diarrhea.

	Models based on SID_3_ and SIG							Models based on SID_5_ and USI						
Order of entry	Variable	Δ R^2^	Model R^2^	Coeff.	± SE	*p*-value	VIF	Variable	Δ R^2^	Model R^2^	Coeff.	± SE	*p*-value	VIF
Venous blood pH as dependent variable														
	Constant	–	–	7.335	0.019	< 0.001		Constant	–	–	7.324	0.02	< 0.001	–
1	SIG	0.539	0.539	0.018	0.0004	< 0.001	1.87	USI	0.532	0.532	0.018	0.0005	< 0.001	1.55
2	SID_3_	0.241	0.780	0.016	0.0005	< 0.001	1.99	SID_5_	0.237	0.766	0.017	0.0005	< 0.001	1.76
3	pCO_2_	0.09	0.871	−0.008	0.0004	< 0.001	2.06	pCO_2_	0.094	0.860	−0.008	0.0004	< 0.001	2.04
4	A_tot_	0.095	0.966	−0.012	0.001	< 0.001	1.21	A_tot_	0.099	0.961	−0.012	0.001	< 0.001	1.21
Actual bicarbonate concentration as dependent variable														
	Constant	–	–	−0.745	0.330	0.026	–	Constant			−1.255	0.325	< 0.001	
1	pCO_2_	0.556	0.556	0.099	0.007	< 0.001	2.06	pCO_2_	0.556	0.556	0.101	0.007	< 0.001	2.04
2	SIG	0.173	0.729	0.790	0.007	< 0.001	1.89	USI	0.178	0.733	0.784	0.008	< 0.001	1.55
3	SID_3_	0.223	0.951	0.813	0.008	< 0.001	1.99	SID_5_	0.219	0.952	0.805	0.008	< 0.001	1.76
4	A_tot_	0.046	0.997	−0.484	0.011	< 0.001	1.21	A_tot_	0.045	0.997	−0.483	0.011	< 0.001	1.21

Δ R^2^, increase in R^2^ caused by the addition of the variable to the multivariable linear regression model; Coeff., coefficient; SE, standard error; VIF, variance inflation factor; SID_3_, strong ion difference calculated from three strong ions (Na^+^, K^+^, Cl^−^); SID_5_, strong ion difference calculated from five strong ions (Na^+^, K^+^, Ca^2+^, Cl^−^, L-lactate^−^); SIG, strong ion gap; USI, unidentified strong ions.

In order to further assess associations with venous blood pH, plasma or serum concentrations of L-lactate, phosphorus, total protein, sodium, potassium as well as pCO_2_ were entered into an additional multivariable stepwise linear regression model. Plasma chloride concentration as well as serum urea and creatinine concentrations were not entered because of their association with plasma sodium and serum inorganic phosphorus concentrations, respectively (Supplementary Table S1). Inorganic phosphorus, pCO_2_, and serum total protein explained 30.2, 10.2, and 2.9% of the variation of venous blood pH, respectively (Table 3).

**Table 3:** Results of a multivariable stepwise linear regression model for predicting venous blood pH by means of variables of clinical pathology in 118 diarrheic calves (1–5 months of age) with Eimeria-associated diarrhea.

Order of entry	Variable^1^	∆R^2^	Model R^2^	Coefficient	± SE	*p*-value	VIF
	Constant			7.313	0.076	< 0.001	–
1	Phosphorus	0.302	0.302	−0.043	0.008	< 0.001	1.328
2	pCO_2_	0.102	0.403	0.005	0.001	< 0.001	1.023
3	Total protein	0.029	0.432	−0.002	0.001	0.017	1.348

1 Plasma L-Lactate, sodium, and potassium concentration were eliminated at the α = 5% level.

pCO_2_, partial pressure of carbon dioxide; ∆R^2^, change of R^2^ value after adding of respective variable to the multivariable regression model; VIF, Variance inflation factor.

### 3.5 Treatment and outcome

Preadmission treatments were reported to have been administered to 100 calves (84.7%), including analgesic drugs (61 calves), antibiotics (62 calves), and coccidiostatic agents (57 calves). Administration of intravenous fluids was documented in 19 calves (16.1%) at variable time points before hospital admission; however, none of these calves received intravenous fluids on the day of hospitalization.

During hospitalization, a total of 86 calves (72.9%) were treated with intravenous fluids containing 0.9% NaCl and if indicated hypertonic glucose (in concentrations of 10 to 40%) and sodium bicarbonate solutions (in concentrations of 1.4 to 8.4%). Oral electrolyte solutions were offered to 83 calves (70.3%). Intraruminal administration of sodium chloride or sodium bicarbonate (in amounts of 20 to 50 g) was performed in 31 (26.3%) and 22 calves (18.6%), respectively. Sodium salts were dissolved in variable volumes of tap water and administered as hypertonic solutions. All calves had access to water and a salt lick stone.

Considering pretreatment activities before hospitalization, coccidiostatic agents (diclazuril or toltrazuril) were administered orally in 42 (35.6%) of calves. Administration of parenteral antibiotics were continued or initiated in 79 calves (66.9%) due to presence of concurrent bacterial infection, suspected septicemia or fever. Additional supportive treatment consisted of administration of non-steroidal drugs in 112 calves (94.9%). Five calves received a blood transfusion, and 16 calves with persistent tenesmus were administered epidural anesthesia using 98% ethanol based on a previously published protocol (39). Surgical intervention due to umbilical herniation or signs of an acute abdominal emergency was performed in 5 calves.

The overall survival rate of calves of this study population was 60.2% (*n* = 71). The median duration (Q_1_/Q_3_) of hospitalization in these 71 calves was 15 (10/20) days. A total of 47 calves did not survive to hospital discharge of which 40 calves were euthanized and 7 calves died spontaneously. Reasons for euthanasia were not stated in most of the records. However, the presumed reason was most frequently ongoing depression and a concomitant lack of improvement of treatment measures (*n* = 22) and/or a massive deterioration of general condition (*n* = 17). A total of 12 calves were euthanized due to occurrence of an acute abdominal emergency. Presence of neurologic symptoms were only documented in one out 47 calves with a negative outcome, which had a postmortem diagnosis of cortical cerebral necrosis.

### 3.6 Prognostic utility of clinical and laboratory findings

Associations between specific clinical finding and the outcome of treatment are reported in Table 4. Univariable binary logistic regression analysis indicated that impairments of ability to stand, presence of enophthalmos, increased skin tent duration, and poor body condition were associated with a higher risk for NO. Most clinicopathologic variables were similar for NO and PO calves. However, calves with NO had lower plasma sodium, chloride and ionized calcium concentrations, lower venous blood oxygen tension, as well as higher leukocyte counts and serum urea concentrations (Table 5). Measured and pH-corrected ionized calcium concentrations (calculated using Equation 9) had similar diagnostic accuracy. Therefore, measured ionized calcium concentration was used for multivariable modelling.

**Table 4:** Results of univariable logistic regression analysis of the association of clinical signs with hospital mortality in 118 calves (1–5 months of age) with Eimeria-associated diarrhea.

Variable	Total no.	Category (Score)	No. tested	No. (%) with non-survival	OR	95% CI for OR	*p*-value
Behavior	118	Bright, alert (1)	46	14 (30.4)	Ref.		
		Depressed (2)	55	24 (43.6)	1.8	0.8–4.0	0.17
		Apathetic, comatose (3)	17	9 (52.9)	2.6	0.8–8.1	0.11
Posture	118	Ability to stand (1)	68	17 (25.0)	Ref.		
		Impaired ability to stand (2)	29	18 (62.1)	**4.9**	**1.9–12.4**	**< 0.001**
		Recumbency (3)	21	12 (57.1)	**4.0**	**1.4–11.1**	**0.008**
Enophthalmos	118	None (1)	32	8 (25.0)	Ref.		
		Slight to moderate (2)	63	30 (47.6)	**2.7**	**1.1–7.0**	**0.037**
		Severe (3)	23	9 (39.1)	1.9	0.6–6.1	0.27
Skin tent duration		Normal (1)	28	6 (21.4)	Ref.		
		Slightly/moderately increased (2)	67	32 (47.8)	**3.4**	**1.2–9.3**	**0.020**
	118	Markedly increased (3)	23	9 (39.1)	2.4	0.7–8.1	0.17
Suckling reflex	108	Strong (1)	11	4 (36.4)	Ref.		
		Weak (2)	35	16 (45.7)	1.5	0.4–6.0	0.59
		Absent (3)	62	22 (35.5)	1.0	0.3–3.7	0.96
SIRS	118	No (0)	62	21 (33.9)	Ref.		
		Yes (1)	56	26 (46.4)	1.7	0.8–3.6	0.17
Bloody feces	118	No (0)	23	10 (43.5)	Ref.		
		Yes (1)	95	37 (38.9)	0.8	0.3–2.1	0.69
Tenesmus	118	No (0)	52	20 (38.5)	Ref.		
		Yes (1)	66	27 (40.9)	1.1	0.5–2.3	0.79
Tachycardia (>120 beats/min.)	118	No (0)	89	38 (42.7)	Ref.		
		Yes (1)	29	9 (31.0)	0.6	0.3–1.5	0.27
Tachypnoe (> 36 breaths/min.)	118	No (0)	83	35 (42.2)	Ref.		
		Yes (1)	35	12 (34.3)	0.7	0.3–1.6	0.43
Temperature (Normal from 38.5°C to 39.5°C)	118	Normal (0)	59	24 (40.7)	Ref.		
		Hypothermia (1)	43	19 (44.2)	1.2	0.5–2.6	0.72
		Hyperthermia (2)	16	4 (25.0)	0.5	0.1–1.7	0.26
Body condition	118	Good to moderate (1)	78	24 (30.8)	Ref.		
		Poor (2)	34	19 (55.9)	**2.9**	**1.2–6.5**	**0.013**
		Cachectic (3)	6	4 (66.7)	4.5	0.8–26.3	0.095
Concurrent health problem	118	No (0)	87	33 (37.9)	Ref.		
		Yes (1)	31	14 (45.2)	1.35	0.6–3.1	0.48

Ref., Reference value; SIRS, systemic inflammatory response syndrome; OR, Odds ratio; 95% CI for OR, 95% confidence interval for odds ratio. Statistically significant odds ratios and associated p-values are highlighted by bold type.

**Table 5:** Median and interquartile ranges of selected laboratory variables stratified by the outcome of treatment in 118 calves (1–5 months of age) with Eimeria-associated diarrhea.

	Calves with positive outcome (*n* = 71)	Calves with negative outcome (*n* = 47)	
Variable	$\tilde{x\;}$ (Q_1_/Q_3_)	$\tilde{x\;}$ (Q_1_/Q_3_)	*p*-value
Henderson-Hasselbalch acid–base model			
pH	7.322 (7.215/7.373)	7.288 (7.199/7.353)	0.38
pCO_2_ (mm Hg)	45.1 (40.8/48.8)	43.7 (40.2/47.4)	0.26
pO_2_ (mm Hg)	38.3 (33.4/43.9)	35.9 (28.9/40.1)	**0.040**
HCO_3_^−^ (mmol/L)	21.6 (15.8/27.0)	19.4 (15.3/24.4)	0.18
BE(B) (mmol/L)	−3.7 (−11.4/1.8)	−6.3 (−11.6/−0.9)	0.20
AG (mEq/L)	13.5 (9.9/19.7)	15.8 (11.8/20.4)	0.51
SID acid–base model			
A_tot_ (mmol/L)	19.4 (16.9/22.1)	18.5 (16.2/21.1)	0.18
A^−^ (mEq/L)	11.9 (10.4/13.2)	11.2 (10.0/12.4)	0.101
SID_3_ (mEq/L)	36.5 (33.9/39.2)	34.6 (32.3/38.0)	0.14
SID_5_ (mEq/L)	36.7 (33.0/40.0)	34.8 (32.8/37.2)	0.056
SID_eff_ (mEq/L)	34.2 (27.5/39.1)	30.4 (27.2/35.7)	0.063
USI (mEq/L)	−0.9 (−6.1/1.4)	−3.0 (−7.6/0.8)	0.40
SIG (mEq/L)	−0.8 (−6.7/1.4)	−4.8 (−8.5/1.1)	0.22
Electrolytes			
Na^+^ (mmol/L)	119.2 (110.2/126.9)	112.2 (106.2/118.3)	**0.008**
K^+^ (mmol/L)	4.9 (4.1/5.8)	5.0 (4.1/6.2)	0.83
Cl^−^ (mmol/L)	88 (81/97)	82 (78/89)	**0.014**
Cl_c_^−^ (mmol/L)	105 (102/108)	105 (102/108)	0.66
Ca^2+^ (mmol/L)	1.14 (1.09/1.21)	1.10 (1.02/1.14)	**0.003**
Ca^2+^_7.40_ (mmol/L)	1.08 (1.00/1.16)	1.0 (0.94/1.09)	**0.003**
Biochemical analysis			
L-lactate (mmol/L)	2.5 (1.6/3.9)	3.4 (1.7/4.6)	0.13
Glucose (mmol/L)	7.0 (5.5/8.5)	6.8 (5.4/9.1)	0.82
Total protein (g/L)	56.5 (49.2/64.4)	53.9 (47.2/ 61.4)	0.18
Albumin (g/L)	30.8 (27.8/37.4)	29.7 (24.8/32.7)	**0.024**
Globulin (g/L)	25.5 (20.4/28.7)	25.4 (22.3/29.0)	0.88
Phosphorus (mmol/L)	2.7 (2.2/3.4)	2.9 (2.2/3.8)	0.40
Urea (mmol/L)	11.4 (4.5/23.2)	16.9 (9.2/27.3)	**0.041**
Creatinine (μmol/L)	149 (105/213)	191 (113/299)	0.22
Hematologic analysis			
PCV (%)	39.4 (33.6/47.2)	37.4 (31.7/44.1)	0.39
Hemoglobin (g/dL)	11.7 (10.4/13.8)	12.2 (10.3/14.4)	0.95
Leukocytes (G/L)	9.7 (8.0/12.9)	13.8 (8.8/22.2)	**0.010**
Thrombocytes (G/L)	789 (635/1198)	806 (619/1057)	0.92

See Table 1 for reference ranges and abbreviations. *P*-values indicating a statistically significant difference are highlighted by bold type.

**Figure 4:**
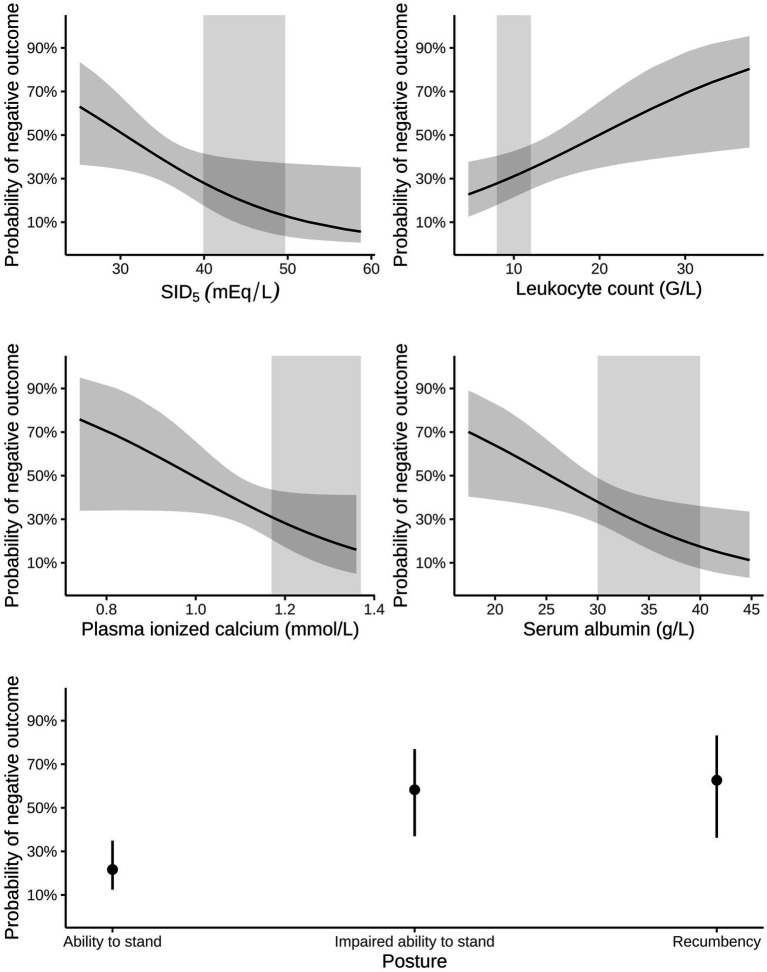
Probability of a negative outcome (i.e., death or euthanasia during hospitalization) in 118 calves (1–5 months of age) with Eimeria-associated diarrhea predicted by leukocyte count, plasma ionized calcium, serum albumin, strong ion difference calculated from five strong ions (SID_5_) and categories of posture/ability to stand. The final choice of predictors was determined by a best subset—automated model selection and multimodel inference approach. Dark, grey-shaded horizontal areas indicate the 95% confidence interval of predicted probabilities, whereas light, grey-shaded vertical areas represent the reference range of respective variables.

Multivariable logistic regression analysis indicated that mortality was associated with impairment of ability to stand, higher leukocyte concentration, and lower concentrations of plasma ionized calcium and serum albumin, as well as decreased plasma SID_5_ (Table 6 and Figure 4)_._ The area under the ROC curve of this model was 0.82 (95% CI: 0.74–0.90, *p* < 0.001) and the resulting sensitivity, specificity, PPV and NPV at the optimal probability cut-off of 0.35 was 78.7% (95% CI: 65.1–88.0%), 71.8% (95% CI: 60.5–81.0%), 64.9% (95% CI: 51.9–76.0%), and 83.6% (95% CI: 72.4–90.8%), respectively. Presence of concurrent health problems was not considered a confounder for this multivariable model.

**Table 6:** Multivariable binary logistic regression models for predicting a negative outcome of therapy during hospitalization by means of clinical and laboratory variables in 118 calves (1–5 months of age) with Eimeria-associated diarrhea.

Variable	Coefficient	SE	Odds ratio	95% CI for odds ratio	*p*-value
Intercept	9.61	3.88			0.013
Posture					
Ability to stand	Reference				
Impaired ability to stand	1.62	0.57	5.05	1.66–15.42	0.004
Recumbency	1.80	0.69	6.06	1.58–23.24	0.009
Leukocytes	0.080	0.033	1.08	1.02–1.16	0.016
Ionized Calcium	−4.52	2.45	0.011	0.00009–1.3222	0.065
Albumin	−0.106	0.046	0.90	0.82–0.98	0.020
SID_5_	−0.099	0.048	0.91	0.82–0.99	0.037

Predictors were identified by means of a best subset-automated model selection and multimodel inference approach.

Hosmer-Lemeshow goodness of fit *X*^2^ = 4.7, df = 8, *p* = 0.79.

**Figure 5:**
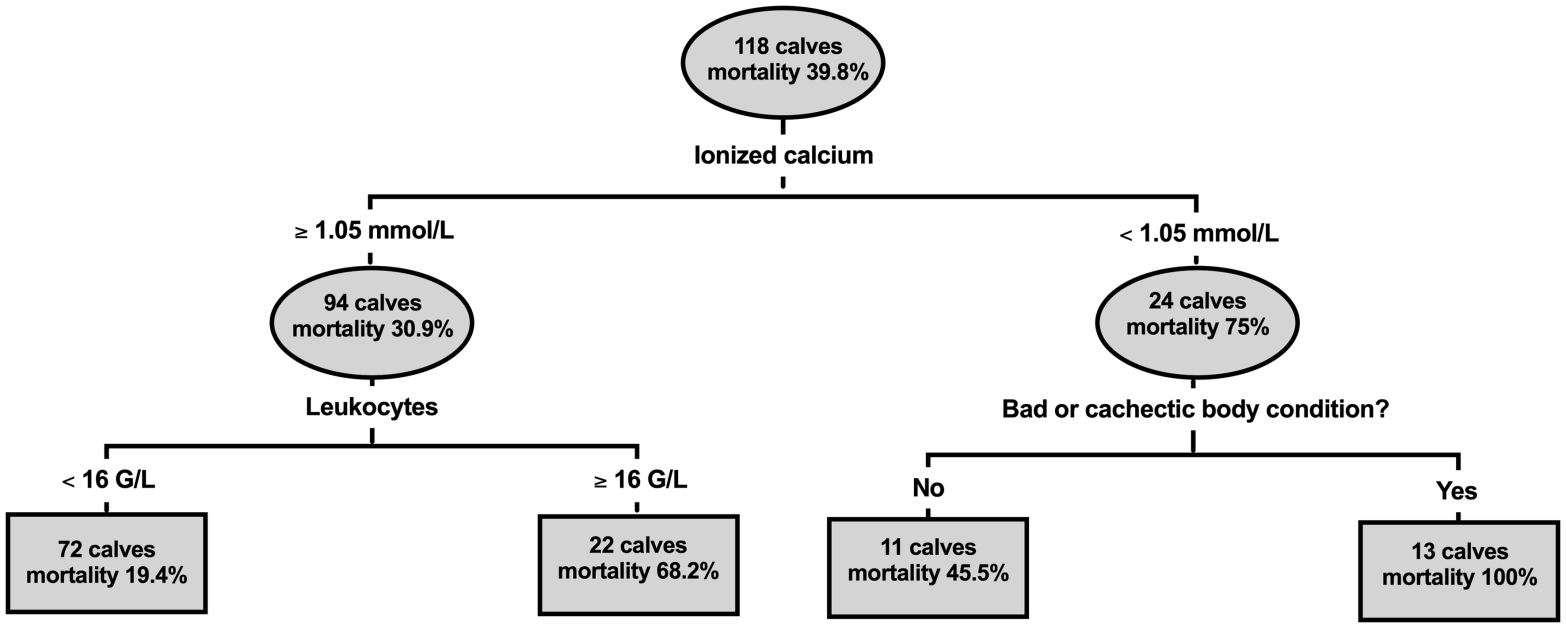
Classification and regression tree illustrating the association of ionized calcium concentration, leukocyte count, and body condition with hospital mortality in 118 calves (1–5 months of age) with Eimeria-associated diarrhea. Lines leaving the oval identify a study variable and its cut-off value that was identified as a significant predictor for a negative outcome of therapy. Branches to the left indicate a respective subgroup with a lower mortality rate, whereas branches to the right indicates subgroups with a higher mortality rate. Squares represent subgroups of calves that are not further subdivided by means of a statistically significant predictor. The tree illustrates that hospital mortality was associated with a plasma ionized calcium concentration < 1.05 mmol/L on admission. In affected calves, mortality was further associated with a poor or cachectic body condition. In contrast in calves with initial plasma calcium concentration > 1.05 mmol/L, initial leukocyte counts >16 G/L were associated with a higher risk for non-survival.

Results of the classification tree analysis are illustrated by Figure 5. This analysis indicated that NO was associated with plasma ionized calcium concentrations <1.05 mmol/L on admission. In those calves, mortality was further associated with a poor or cachectic body condition. In contrast in calves with initial plasma calcium concentration > 1.05 mmol/L, initial leukocyte counts >16 × 10^9^ cells/L were associated with a higher risk for a NO. Based on predicted values, the area under the ROC curve of this model was 0.75 (95% CI: 0.65–0.85; *p* < 0.001). The sensitivity, specificity, PPV, NPV for predicting a negative outcome of this model was 59.6% (95% CI: 45.3–72.4%), 90.1% (95% CI: 81.0–95.1%), 80% (95% CI: 64.1–90%), and 77.1% (95% CI: 67–84.8%), respectively.

## 4 Discussion

Central findings of this retrospective study indicate that electrolyte and acid–base derangements are common in critically ill calves with Eimeria-associated diarrhea. Of interest is the finding that 60.2% of calves had metabolic acidosis (*c*HCO_3_^−^ < 23 mmol/L) and 57.6% of calves were acidemic (venous blood pH < 7.33), which is in contrast to experimental studies where only mild decrements of plasma bicarbonate concentrations and venous blood pH were observed (17). Our data further indicate that acidemia in affected calves was associated with hyperphosphatemia, hyper-L-lactatemia, decreased measured strong ion difference and an increase of strong ion gap. Especially hyperphosphatemia was found as an important predictor for decrements of venous blood pH (Table 3). Hyperphosphatemia was likely the result of dehydration, decreased glomerular filtration rate, and tissue hypoxia based on findings in calves with neonatal diarrhea (10, 11), calves with acute abdominal emergencies (31), and cows with right-sided displacement of the abomasum or abomasal volvulus (40). The association between hyperphosphatemia and decrements of venous blood pH therefore indicates that dehydration is a major contributor to acidemia in calves with clinical coccidiosis. Clinical dehydration was documented in 82.2% of calves of this study population, although serum protein profiles, as well as hematocrit and hemoglobin concentrations were not indicative for profound hemoconcentration in many animals. Variable changes of hematocrit and hemoglobin concentrations were seen in calves after experimental infection with *E. bovis* and *zuernii* (3, 4, 18, 21), and it is likely that the effect of hemoconcentration was masked by intestinal losses of blood and proteins in calves of the present study.

Interestingly, the multivariable model for the prediction of venous blood pH based on plasma L-lactate, pCO_2_, serum total protein and phosphorus, and plasma concentrations of sodium and potassium explained only 43.2% of the variation of venous blood pH. Furthermore, the quantitative physicochemical analysis of acid–base balance indicated the presence of unidentified strong anions in many calves. In this context it is of relevance that the USI concentration was identified as the most important predictor of venous blood pH in the multivariable model based on SID_5,_ pCO_2_, A_tot_, and USI (Table 2). Notable increases of unidentified strong ions were also previously reported in calves with neonatal diarrhea and surgical abdominal emergencies (10, 11, 31, 41). The nature of these anions in calves of the present study remains unknown but are likely to be uremic anions based on studies in calves with neonatal diarrhea (10, 11) and adult cattle (28) and by the coefficients of correlation between USI and parameters of renal function and hydration status in the present study such as inorganic phosphorus (*r_s_* = −0.73), urea (*r_s_* = −0.60), and creatinine (*r_s_* = −0.65). Hyper-D-lactatemia was considered an unlikely contributor to USI concentration in this study based on plasma or serum D-lactate concentrations from 27 calves. Small increases in ß-hydroxybutyrate concentrations have been reported in dehydrated neonatal calves with a negative nutrient balance (42) and in older calves during the weaning process (43); however, serum ß-hydroxybutyrate was measured in a small subset of animals in this study where it did not represent a quantitatively important contributor to USI concentration. Increased concentrations of urate, oxalate and sulfate were reported in human patients with renal failure (44–46). For that reason, it is likely that uremic anions accounted for the increase of plasma USI because of dehydration and decreased glomerular filtration rate. Especially both hyperphosphatemia and hypersulfatemia are present in human patients with renal failure (44, 45). The positive association between plasma inorganic phosphorus and sulfate concentrations provides a plausible explanation why decrements of venous blood pH were best predicted by increased inorganic phosphorus concentrations in the present study (Table 3) as previously reported for dehydrated neonatal diarrheic calves with hyperkalemia (11).

Clinical infections with *Eimeria zuernii* or *Eimeria bovis* can cause severe damage to large intestinal mucosa, with the colon and caecum being particularly affected. Lesions are characterized by a diphtheritic typhlitis and colitis which can include haemorrhages in the mucosa and submucosa, destruction and loss of epithelial cells with subsequent exposure of blood vessels in the lamina propria, and formation of crypt abscesses and intraluminal diphtheritic membranes (47–50). Profound hyponatremia and hypochloremia has been frequently documented in critically ill calves with experimentally-induced and naturally acquired coccidiosis (4, 17, 22–24), goats with naturally acquired coccidiosis (51), and horses with Equine neorickettsiosis, an infectious disease of the large intestine (52); this is in marked contrast to calves with neonatal diarrhea that appears to be a small intestinal infection with mild to moderate hyponatremia (10, 53). Experimental Eimeria infection impaired sodium and chloride absorption in the rat large intestine (54) and impaired sodium absorption in the calf large intestine (24) (chloride absorption was not studied). Hyponatremia and accompanying hypochloremia in calves with Eimeriosis is therefore most likely due to decreased absorption of sodium and chloride by the proximal large intestine and plasma loss into the large intestinal lumen due to morphologic damage to the mucosa, rather than active hypersecretion (24). The strong positive correlation between plasma sodium and chloride concentrations in calves of the present study (*r_s_* = 0.90) was higher than previously reported coefficients of 0.64 (*n* = 55) (41) and 0.75 (*n* = 806) (10) for neonatal calves with diarrhea. This indicates that critically ill calves with Eimeriosis typically experience similar reductions of plasma sodium and chloride concentrations and therefore milder decrements of SID_3_ when compared to neonatal calves with diarrhea. However, derangements of SID_3_ were still seen and associated with changes of venous blood pH and bicarbonate concentration (Figure 2; Table 2). Beside pathophysiological consequences from diarrhea, the observed decrements of SID_3_ could be additionally related to differential compartment shifts of sodium and chloride ions due to endotoxemia or sepsis (55, 56), or a compensatory increase of chloride due to hypoalbuminemia to conserve electroneutrality (57).

Encephalopathy and neurologic alterations have been documented as rare complications of marked hyponatremia in foals (58, 59). Furthermore, demyelinating brain lesions including pontine myelinolysis were reported in humans and rats after rapid correction of chronic and severe hyponatremia with reported mean plasma concentrations of <100 mmol/L (60, 61). In calves, reports about the clinical picture of nervous coccidiosis exist (mainly from North America in calves aged 6 to 12 months of age), which is characterized by ataxia, tremor, opisthotonos, strabismus, foaming and tonic–clonic seizures that can be induced by stress (62–64). A clear causal relationship between hyponatremia and neurologic signs in affected animals has not been proven and other electrolyte imbalances and the presence of a neurotoxins were discussed to be involved (65–67). The clinical picture of nervous coccidiosis is generally not seen in our hospital population. Of interest is however the finding, that calves with NO had significantly lower plasma sodium concentrations in the present study than calves with PO. However, signs of CNS involvement were not documented in calves with NO (except in one case with a post-mortem diagnosis of cerebrocortical necrosis). Furthermore, hyponatremia was not identified as a risk factor for NO during multivariable binary logistic regression and classification tree analysis. For those reasons, the association between hyponatremia and mortality was likely an expression of disease severity and/or duration. Nevertheless, because pontine myelinolysis is associated with rapid increases in plasma sodium concentration in animals with severe hyponatremia (< 120 mmol/L), a goal of treatment of recumbent calves with coccidosis should be to slowly increase plasma sodium concentration as outlined elsewhere (68, 69). Current recommendations are to increase serum sodium concentration by no more than 8 (mmol/L)/day in large animals with chronic hyponatremia (68). For practical purposes (especially in situations where measurement of plasma or serum sodium concentrations is impossible), we recommend rehydrating calves that need i.v. fluids with isotonic solutions and avoiding the use of hypertonic sodium solutions. Acetated or lactated ringer solution might provide some advantage in this context, as these solutions have a lower sodium concentration (140 mmol/L and 130 mmol/L, respectively) than 0.9% NaCl solutions (sodium concentration, 154 mmol/L). However, this is an area where more specific research is needed.

Experimental studies reported an increase of fecal losses of potassium in diarrheic calves after experimental inoculation of *Eimeria bovis* oocysts (24), but also markedly increased plasma potassium concentrations that were especially documented during an advanced disease stage (22). Hyperkalemia and hypokalemia were found in the present study with a similar prevalence as previously reported for calves with neonatal diarrhea in referral hospital settings (11, 70, 71). In neonatal diarrheic calves, the occurrence of hyperkalemia was historically related to an acidemia-induced dysregulation of potassium homeostasis between the intra- and extracellular space (72). Recent research based on large-scale studies indicated that the presence of hyperkalemia depends on the nature of an existing acidosis, with hyper-D-lactatemia being rarely associated with higher-than-normal plasma potassium concentrations (11, 70). More importantly, hyperphosphatemia and azotemia were identified as main risk factors for hyperkalemia, strongly indicating that dehydration and a concomitant decrease of glomerular filtration rate are central pathophysiological events (11). Similar associations were found in the present study (Figure 1), although the coefficient of correlation between venous blood pH and plasma potassium concentrations (*r_s_* = −0.44) was higher than reported for neonatal diarrheic calves (*r_s_* = −0.21; (11)), most probably related to the finding that hyper-D-lactatemia did not appear to play a significant role in this study population.

In neonatal diarrheic calves, hyperkalemia is associated with a clinical picture characterized by impairments of ability to stand, cyanosis, profound dehydration and cardiac arrhythmias (70). Clinical effects are related to an impaired neuromuscular excitability which can lead to skeletal muscle weakness and cardiac conduction abnormalities that can manifest in the electrocardiogram as widening of the QRS-complex, occurrence of large and spiked T-waves, decreased amplitude or disappearance of P waves, and ventricular escape rhythms and arrhythmias (33, 70). Likely, hyperkalemia was also of clinical relevance in calves of this study population as the clinical effects of hyperkalemia are known to be exacerbated by hyponatremia (33), which had a high prevalence of 90%. This could explain why hyponatremia, hyperkalemia, and a concomitant increase of K^+^/Na^+^-ratio was associated with impairments of posture/ability to stand. Unfortunately, ECG recordings were not available for the calves in the study reported here.

In the present study, hyperglycemia was seen in 71.2% of calves, whereas hypoglycemia was not observed. These findings suggest that administration of glucose containing infusion solutions is not needed in most calves with clinical coccidiosis requiring intravenous fluid therapy. The observed prevalence of hyperglycemia is in marked contrast to studies in neonatal diarrheic calves, where hypoglycemia is much more frequently observed (13, 73). A previous study (73) compared serum glucose concentration between a study population of neonatal diarrheic calves (*n* = 283) with those of a study population of older calves with diarrhea (aged 1 to 5 months; *n* = 153). In that study hyperglycemia was seen in only 0.7% of calves with neonatal diarrhea, but in 75% of older calves with diarrhea (73). In the latter group, stress reactions due to severe dehydration were considered as an explanation for hyperglycemia based on positive correlations between plasma glucose and serum urea or creatinine concentrations (which was also seen in this study population; see Supplementary Table S1). The authors speculated that a similar hyperglycemic response is not possible in neonatal diarrheic calves because of limited glycogen reserves or prolonged anorexia. Hyperglycemia was also documented in hospitalized infants with diarrhea where it is also associated with a stress reaction associated with hypovolemia (74, 75). It is well accepted that hyperglycemia is beneficial in hypovolemic animals as it promotes translocation of intracellular fluid to the extracellular space, thereby increasing plasma volume, venous return, and cardiac output (76, 77).

Multivariable binary logistic regression analysis indicated that mortality was associated with impairment of ability to stand, leukocytosis, hypocalcemia, hypoalbuminemia, as well as decreased plasma SID_5_. Furthermore, classification tree analysis revealed that hospital mortality was associated with plasma ionized calcium concentrations <1.05 mmol/L and a poor or cachectic body condition. In calves with initial plasma calcium concentration > 1.05 mmol/L, initial leukocyte counts >16 G/L were additionally associated with a higher risk for NO. The association of these identified variables with hospital mortality might be interpreted as an expression of a systemic inflammatory response and advanced disease severity.

The disrupted barrier of the intestinal mucosa in severely diseased calves might allow bacteria and endotoxin to enter the bloodstream from the intestine. A connection between intestinal damage and the presence of a systemic inflammatory reaction has been described for coccidiosis in poultry (78, 79). Hypocalcemia is frequently associated with sepsis in human patients (80–82) as in other species (83–85) and was also observed after experimentally induced endotoxemia in adult cattle (86, 87), pigs (88), horses (89), and dogs (90). Adverse effects from endotoxemia and/or sepsis might therefore explain the observed higher risk for NO in hypocalcemic calves and also provide an explanation why leukocyte concentrations >16 × 10^9^ cells/L were associated with mortality in calves with an initial plasma ionized calcium concentration ≥ 1.05 mmol/L. Hypocalcemia in septic patients was also reported to be associated with an increased risk for organ dysfunction and mortality in humans and dogs (81–83, 91, 92). The link between hypocalcemia and sepsis or endotoxemia is not completely understood, but low parathyroid hormone concentrations due to damage to the parathyroid gland or suppression of the gland by cytokines, reduced vitamin D synthesis, sequestration of calcium within the gastrointestinal tract, and calcium chelation with L-lactate are discussed (86, 90, 93–96). In the present study, only 20.3% of calves had *c*Ca < 1.05 mmol/L, which was identified as a clinically useful cut-point value during classification tree analysis. In this context it needs to be considered that the plasma ionized calcium fraction in calves is dependent on blood pH, but also on plasma concentrations of chloride and L-lactate (32). However, the measured plasma ionized calcium concentration represented the biologically active form of calcium (32) and corrective equations were therefore not used for multivariable modelling.

Another condition that was significantly associated with hospital mortality was hypoalbuminemia. This finding can be additionally explained by disease severity in terms of large intestinal organ damage and dysfunction of the blood-gut barrier and a concomitant loss of blood proteins. Furthermore, albumin is known as a negative acute phase protein and hypoalbuminemia could therefore also be fostered by a systemic inflammatory state and a concomitant acute phase reaction resulting in extravasation and decreased hepatic albumin synthesis (97, 98). Reduced growth rate, weight gain and performance are well-known consequences of coccidiosis in calves (3, 18, 21), and markedly reduced body condition was also documented in 34% of calves of the present study. For that reason, it is also conceivable that decrements of serum albumin concentration were also favored by a catabolic state or the combined presence of malnutrition and inflammation (97).

Advanced disease severity further explains the association of NO with alterations of posture/ability to stand and reduced body condition. A poor or cachectic body condition was also identified as a negative prognostic factor in neonatal diarrheic calves (13) and malnutrition is also a known risk factor for fatal diarrhea in children (99, 100).

To the best of our knowledge, this is the first study addressing acid–base imbalances and prognostic factors in a cohort of calves with naturally acquired Eimeria-associated diarrhea to be published. However, the present study has several limitations. A major limitation is the retrospective study design, which is prone for documentation issues and a potential bias related to individual treatment variations or change of laboratory equipment over time. The documented clinical picture of calves was consistent with Eimeriosis and was confirmed by fecal examinations. For that reason, no further diagnostic work-up was performed in the vast majority of animals. Related to the retrospective nature of this study, it can therefore not be ruled-out that concurrent intestinal infections (e.g., due to *Giardia duodenalis* or even *Salmonella* spp.) were additionally present in some single calves. Also, coprological examinations were mostly conducted in-house, which did not always include a differentiation between pathogenic and apathogenic *Eimeria* spp. Furthermore, it needs to be considered that this study was based on a referral hospital population that is usually preselected towards more complicated and severely affected cases and where pre-treatment activities might had an impact on the outcome of our analyses. For that reason, results of this study may not be directly transferable to the situation in ambulatory bovine field practice. Use of a potentially preselected study population was underscored by the presence of concurrent health problems that were documented in 26.3% of calves during the first 48 h of hospitalization. However, concurrent presence of non-gastrointestinal disease, such as pneumonia, was considered unlikely to have impacted serum electrolyte concentrations and acid–base balance as assessed using the physicochemical approach. Furthermore, it is well known that critical illness can predispose calves to secondary health problems (13) and removal of cases with concurrent disease likely would have decreased the external validity of this study. Nevertheless, findings of the present analyses need to be confirmed in future studies that are based on potentially larger study populations and that are ideally performed in a field or on-farm setting.

## 5 Conclusion

Findings of this retrospective study indicate that calves with Eimeria-associated diarrhea can develop profound metabolic derangements including marked hyponatremia, hypochloremia, hyperkalemia, azotemia, and acidemia due to strong ion (metabolic) acidosis. In this study population of hospitalized calves, acidemia was present in 57.6% of cases and was characterized by hyperphosphatemia, hyper-L-lactatemia, and the presence of unidentified strong ions that were associated with indices of altered hydration status and impaired renal function. Results of our analyses suggest that alterations of posture/ability to stand, the finding of a poor or cachectic body condition, hypocalcemia, leukocytosis, decrements of strong ion difference, and hypoalbuminemia should alert clinicians towards a higher risk for non-survival.

## Data Availability

The original contributions presented in the study are included in the article/Supplementary material; further inquiries can be directed to the corresponding author.
