# Supplementary material for: Retrospective evaluation of acid–base imbalances, clinicopathologic alterations, and prognostic factors in hospitalized calves with Eimeria-associated diarrhea
Source: Front Vet Sci. 2025 Jan 6;11:1467583. doi: 10.3389/fvets.2024.1467583 (PMC11743278; doi:10.3389/fvets.2024.1467583)
Supplement: Supplementary file 1 [file Table_1.pdf]

Supplementary Material

Supplementary Table S1. Spearman's coefficients of correlation between selected clinicopathologic findings in 118 calves with Eimeria-associated diarrhea.

	pH	pCO_2	pO_2	HCO_3^-	BE	SID_3	SID_5	SID_{eff}	USI	AG	SIG	Na^+	K^+	Cl^-	Ca^{2+}	L-lac	TP	Alb	P	Urea	Crea	Gluc
**pH**	1.0.																					
**pCO_2**	0.45**	1.0.																				
**pO_2**	0.18NS	0.01NS	1.0.																			
**HCO_3^-**	0.92**	0.72**	0.12NS	1.0.																		
**BE**	0.95**	0.66**	0.16NS	0.99**	1.0.																	
**SID_3**	0.33**	0.31**	-0.16NS	0.37**	0.36**	1.0.																
**SID_5**	0.48**	0.46**	-0.04NS	0.55**	0.54**	0.86**	1.0.															
**SID_{eff}**	0.89**	0.68**	0.13NS	0.97**	0.96**	0.44**	0.62**	1.0.														
**USI**	0.66**	0.47**	0.23*	0.70**	0.70**	-0.21*	-0.09NS	0.67**	1.0.													
**AG**	-0.63**	-0.49**	-0.26*	-0.69**	-0.69**	0.32**	0.08NS	-0.59**	-0.90**	1.0.												
**SIG**	0.67**	0.47**	0.26*	0.71**	0.72**	-0.24*	0.01NS	0.69**	0.95**	-0.95**	1.0.											
**Na^+**	0.20*	0.29**	0.34**	0.28*	0.29*	0.14NS	0.16NS	0.24**	0.21*	-0.27**	0.24**	1.0.										
**K^+**	-0.44**	-0.35**	-0.38**	-0.49**	-0.50**	0.07NS	0.00NS	-0.42**	-0.59**	0.58**	-0.56**	-0.41**	1.0.									
**Cl^-**	0.03NS	0.13NS	0.39**	0.09NS	0.10NS	-0.23*	-0.16NS	0.04NS	0.24*	-0.35**	0.30**	0.90**	-0.37**	1.0.								
**Ca^{2+}**	-0.22*	-0.00NS	0.33**	-0.15NS	-0.13NS	-0.16NS	-0.14NS	-0.13NS	-0.02NS	-0.04NS	0.02NS	0.61**	-0.07NS	0.70**	1.0.							
**L-Lac**	-0.32**	-0.27**	-0.30*	-0.37**	-0.38**	0.21*	-0.20*	-0.35**	-0.35**	0.58**	-0.59**	-0.29**	0.31**	-0.38**	-0.18NS	1.0.						
**TP**	-0.43**	-0.22*	-0.10NS	-0.41**	-0.42**	0.16NS	0.11NS	-0.19*	-0.34**	0.58**	-0.36**	-0.23*	0.35**	-0.24*	0.03NS	0.20*	1.0.					
**Alb**	-0.43**	-0.15NS	-0.10NS	-0.38**	-0.39**	0.20*	0.14NS	-0.19*	-0.36**	0.55**	-0.37**	-0.10NS	0.31**	-0.12NS	0.19*	0.21*	0.87**	1.0.				
**P**	-0.51**	-0.27*	-0.33**	-0.51**	-0.52**	0.27*	0.01NS	-0.44**	-0.73**	0.76**	-0.73**	-0.38**	0.56**	-0.44**	-0.23*	0.48**	0.48**	0.45**	1.0.			
**Urea**	-0.46**	-0.39**	-0.36**	-0.51**	-0.52**	0.12NS	-0.01NS	-0.44**	-0.60**	0.67**	-0.62**	-0.69**	0.49**	-0.69**	-0.51**	0.46**	0.50**	0.41**	0.80**	1.0.		
**Crea**	-0.42**	-0.33**	-0.35**	-0.46**	-0.47**	0.22*	0.09NS	-0.40**	-0.65**	0.70**	-0.65**	-0.52**	0.57**	-0.57**	-0.40**	0.43**	0.44**	0.38**	0.80**	0.83**	1.0.	
**Gluc**	-0.29*	-0.16NS	-0.27*	-0.29*	-0.31**	0.13NS	-0.03NS	-0.23*	-0.33**	0.44**	-0.43**	-0.45**	0.34**	-0.46**	-0.15NS	0.49**	0.32**	0.27*	0.39**	0.45**	0.38**	1.0

** $P < 0.001$, * $P < 0.05$, NS not significant

pCO_2 = partial pressure of carbon dioxide, BE = base excess, AG = anion gap. A_{tot} = concentration of non-volatile weak acids, SID_3 = strong ion difference calculated from three strong ions, SID_5 = strong ion difference calculated from five strong ions, SID_{eff} = effective strong ion difference, USI = unidentified strong ions, SIG = strong ion gap, L-Lac = L-lactate, TP = total protein, Alb = albumin, Crea = creatinine, Gluc = glucose
